# Comparison of results of graft uptake using tragal cartilage perichondrium composite graft versus temporalis fascia in patients undergoing surgery for chronic otitis media – squamous type

**DOI:** 10.1186/s13005-016-0123-z

**Published:** 2016-08-02

**Authors:** S. Khalilullah, Shankar P. Shah, D. Yadav, R. P. Shrivastav, H. Bhattarai

**Affiliations:** 1 Department of ENT & HNS, Government Medical College, Chandigarh, India; 2 Department of ORL & HNS, BPKIHS, Dharan, Nepal; 3 Department of ORL&HNS, PAHS, Kathmandu, Nepal; 4 Department of ORL&HNS, TUTH, Kathmandu, Nepal

**Keywords:** Chronic otitis media-squamous type, Tragal cartilage perichondrium composite graft, Postero-superior retraction pocket

## Abstract

**Background:**

To assess, analyze and compare the results of graft uptake using Tragal Cartilage-Perichondrium Composite (TCPC) graft with Temporalis Fascia (TF) graft in patients who undergoing surgery for chronic otitis media - squamous type.

**Methods:**

Patients aged 13 years and above with diagnosis of chronic otitis media - squamous type undergoing modified radical mastoidectomy, either primary or revision surgery with grafting of tympanic membrane (TM) and patients undergoing excision of postero-superior retraction pocket (PSRP) were included in this study. Patients were divided in two groups: Group A-patients undergoing TCPC graft and Group B -patients undergoing TF graft. Graft uptake results were assessed between 8-12 weeks of surgery.

**Results:**

In both Groups there were 30 patients each. In Group A successful graft uptake was seen in 27 patients (90 %) and failure of graft uptake was seen in 3 patients(10 %). In Group B successful graft uptake was seen in 28 patients (93.3 %) and failure in 2 patients (6.67 %).Out of the total 60 patients, 11 patients had PSRP. All 6 patients with PSRP in Group A had successful graft uptake and no retraction. Among the 5 patients with PSRP in Group B all patients had sucessful graft uptake, however, in 2 patients retraction of the tympanic membrane was seen similar to the preoperative findings.

**Conclusions:**

There was no statistical difference (*p* = 0.433) between the use of temporalis fascia or tragal cartilage perichondrium in patients undergoing surgery for chronic otitis media - squamous type.

## Background

Chronic otitis media (COM) is a not an uncommonly encountered condition by an Otolaryngologist in their day to day practice. According to the WHO the Western Pacific and South-east Asian regions would contribute about 85–90 % of the global burden from Chronic suppurative otitis media (CSOM), with India and China accounting for much of the burden, the former by its high CSOM prevalence rate and the latter by its sheer size [1]. Chronic otitis media is still one of the most common ear diseases in many of the developing countries and the prevalence of squamous type of COM is 3.5 % in Nepal [2].

The management of COM - squamous is mainly surgical. There are different modalities of surgical treatment available, either canal wall up (CWU) or canal wall down (CWD) tympanomastoidectomy depending upon the disease and the patient [3, 4]. Following tympanomastoidectomy, it is usual to graft the middl ear and mastoid cavity. It has been found that when Temporalis fascia is used as graft material, underlying pathological changes occur in the graft particularly when there is a shallow middle ear cavity resulting in diffuse local retraction, cholesteatoma formation and occasional persistent perforation. This leads to recurrent infection [5, 6]. This has led to the use of cartilage, which is a less compliant and a more rigid material that resists resorption and retraction. Cartilage has been shown to reduce recurrent retraction pockets, when used for reconstruction of the posterosuperior quadrant of the tympanic membrane [6]. Cartilage also offers the advantage of higher mechanical stability compared with membranous transplants thus preventing retraction of tympanic membrane in the long run. However, others argue that it may alter the acoustic transfer characteristics of the graft due to the increasing mass and stiffness of the reconstructed tympanic membrane [7].

There are several studies comparing tragal cartilage perichondrium composite graft with temporalis fascia in myringoplasty and tympanoplasty operations. However, there are few studies in the literature comparing TCPC with temporalis fascia in surgery for Chronic otits media – squamous type. Therefore this study was conducted to compare the results of graft uptake using tragal cartilage perichondrium graft versus temporalis fascia in patients undergoing surgery for chronic otitis media - squamous type.

## Methods

A prospective, longitudinal and a comparative study was conducted at the Dept. of ENT & Head and Neck Surgery, Institute of Medicine, Tribhuvan University Teaching Hospital, Kathmandu, Nepal for a period of 18 months, from 1st January 2009 to to 31st June, 2010. The study comprised of 60 patients, 30 in each of two groups (Group A and Group B). Group A included those patients in whom Tragal cartilage - perichondrium composite graft was used and Group B in those Temporalis fascia graft was used following tympanomastoidectomy. Patients of both gender, aged 13 years and above undergoing modified radical mastoidectomy (MRM) either primary or revision surgery and those undergoing excision of posterior-superior retraction pocket (PSRP) were included in the study whereas those with Chronic otitis media squamous with intact pars tensa at the end of surgery (primarily attic disease) were excluded. Written informed consent was obtained from the patient’s legal guardian(s) for publication of this case report and any accompanying images. A copy of the written consent is available for review by the Editor-in-Chief of this journal.

### Pre-operative workup and surgical technique

The patients attending the outpatient department with the diagnosis of COM squamous type and requiring MRM underwent otoscopy, tuning fork tests and preoperative hearing evaluation by pure tone audiogram.The patients were randomized in to two groups using a simple lottery method - Group A those in whom Tragal cartilage - perichondrium composite graft was to be used and Group B those that were to be grafted with Temporalis fascia. All the surgeries were performed by two senior professors (RPS and HB) in the Otology unit of the department.

### Surgical technique

**Fig. 1:**
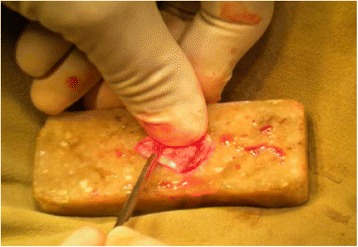
Harvested tragal cartilage with intact perichondrium

**Fig. 2:**
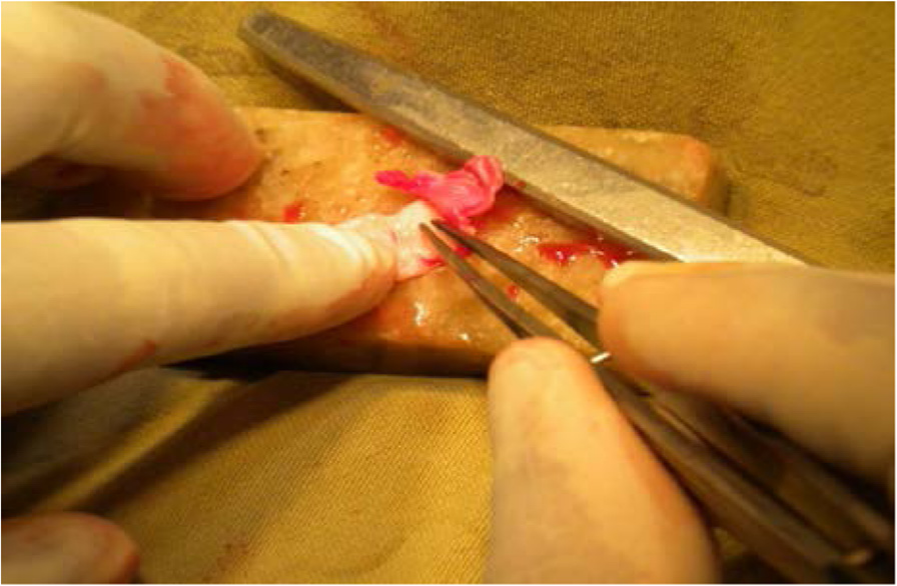
Perichondrium from the anterior aspect of the tragal cartilage being removed

**Fig. 3:**
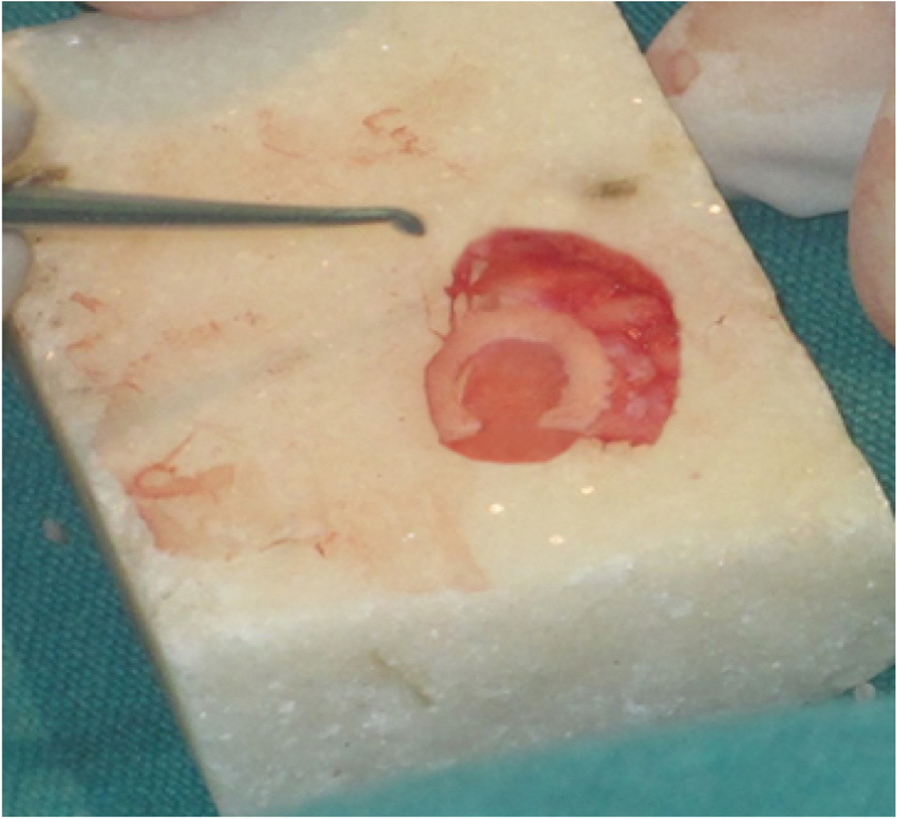
The final TCPC graft with removed central cartilage

The approach was either end-aural, extended endaural or post-aural. In patients with chronic otitis media squamous type, modified radical mastoidectomy was done and either TCPC grafting (Figs. 1, 2 and 3) or temporalis fascia grafting was done as per the randomisation. In patients with posterior superior retraction pocket, after excision of the retraction pocket either TCPC graft or temporalis fascia graft was used. Randomization was done using simple lottery method from a box of coupons kept in a box in the Operation theatre. After the surgery and just before placing the graft the resident on the floor was asked to pick out the coupon and graft- either fascia or TCPC was done as per the lottery.

### TCPC graft

An incision along the free edge of medial side of the tragus was performed leaving 2 millimetre of cartilage in dome of tragus for cosmesis and the subcutaneous tissue was dissected to the lateral border of the cartilage and its perichondrium. The cartilage was then widely exposed on both its lateral and medial surfaces and then harvested with its attached perichondrium; the donor site was then closed. The harvested graft was about 12 mm in diameter andcircular in shape but was cut down and tailored to the size of the perforation or retraction pocket. An ear speculum (5-6 mm in diameter) was placed against the cartilage to mark the piece of cartilage to be removed and a surgical blade was used in order to cut through cartilage alone leaving the perichondrium intact on the posterior aspect of the cartilage. The cut central circular piece of cartilage was removed bygentle dissection. Then a further strip of cartilage was removed at one end of the cartilage in continuation to the ring of cartilage with a gap in the ring to accommodate the handle of malleus/remnant ossicle as shown in figure (Figs. 1, 2 and 3).

### Temporalis fascia graft

Fascia of the ipsilateral temporalis muscle was harvested from the same incision site in post‐aural, endaural incision or extended endaural as approached to the middle ear. Any remaining muscle fibers were removed from the fascia bluntly and was cut into an appropriate size after it had dried up. After modified radical mastoidectomy or excision of the postero-superior retraction pocket, the graft was tucked under the remnant tympanic membrane as an underlay technique.

Oral ciprofloxacin were prescribed in postoperative period for 10 days. Skin sutures were removed on sixth postoperative day of surgery. Bismuth Iodoform Paraffin Paste pack that was kept in the external auditory canal was removed on tenth post operative day. Follow up was done in 6th – 8th weeks and 12th weeks postoperatively by the two senior professors performing the surgery. Graft uptake evaluation was performed after 12 weeks of surgery in the completely dried ear or with a minimal discharge from mastoid cavity but a completely healed middle ear. Residual perforation of any size was taken as failure of graft up take. Statistical analysis was done using SPSS Software (16.0). Chi square test and Fischer’s exact test was applied between the two groups for the test of significance. *p*-value < 0.05 was taken as significant.

## Results

A total of 64 patients were enrolled in our study, 4 cases were excluded due to irregularities in follow up. In Group A (TCPC graft), 24 patients underwent modified radical mastoidectomy and 6 patients underwent excision of postero -superior retraction pocket whereas in Group B (Temporalis fascia graft) 25 patients underwent modified radical mastoidectomy and 5 excision of postero- superior retraction pocket. The average age of patients in TCPC group was 23.21 years where as in temporalis fascia group it was 23.93 years. In Group A there were 18(60 %) male patients and 12 (40 %) female patients (male to female ratio was 1.5:1) whereas in Group B there were 16 (53.3 %) male and 14 (46.7 %) female (male to female ratio was 1.1:1).

Among the 30 patients in Group A, graft uptake was seen in twenty seven patient (90 %). In Group B among 30 patients, 28 patients (93.3 %) had graft uptake (Table 1). Out of 60 patients, 11 patients had excision of postero-superior retraction pocket. Six patients from Group A and 5 from Group B. All 6 patients with TCPC grafting had graft uptake in postoperative period. None of the patient had postoperative graft failure or retraction. In Group B 5 patients underwent Temporalis fascia grafting. Though all of them had graft uptake but 2 patients had graft retraction. Although our study showed better graft uptake using temporalis fascia graft (Table 1), no statistical difference in the graft uptake was seen between these two groups (*p* = 0.433).

**Table 1:** Graft uptake in patients with TCPC graft (GROUP A) and temporalis fascia (GROUP B)

Type of Graft	Total no of cases	Uptake	Failure
TCPC Graft (GROUP A)	30	27 (90 %)	3 (10 %)
Temporalis fascia graft - GROUP B	30	28 (93.3 %)	2 (6.7 %)

Fischer’s Test *p* = 0.433

## Discussion

The objective of this study was to assess, analyze and compare the results of graft uptake between TCPC graft and temporalis fascia graft in patients undergoing surgery for COM squamous type following either modified radical mastoidectomy or excision of postero-superior retraction pocket. Temporalis fascia graft is thin and soft so it cannot resist retraction in cases of negative middle ear pressure. It also has a tendency to shrink after final positioning in the middle ear. Various authors claim that cartilage graft is a better grafting material than temporalis fascia graft. [6, 7] Tragal cartilage perichondrium composite graft used in this study has advantage over other graft types because it has both soft and rigid components and is also malleable and tense.

There are many studies where TCPC graft has been compared to temporalis fascia graft in myringoplasty. However, there are very few studies that have compared TCPC graft with temporalis fascia graft in modified radical mastoidectomy. In our study 30 patients underwent TCPC graft. In 27 patients there was successful graft uptake (90 %) while 3 patients had graft failure (10 %). Thirty (30 %) patients underwent temporalis fascia graft. In 28 patients there was successful graft uptake (93.3 %), while there were 2 patients with graft failure (6.7 %).

Mansour et al [8] in 2006 reported a success rate of 100 % with TCPC graft. In his study tympanoplasty with canal up mastoidectomy was performed in 10 patients and ossiculoplasty was performed in 8 patients using the TCPC graft. In our study all 6 patients with PSRP who had undergone TCPC had graft uptake (100 %) and there was no retraction. This is comparable to the results of Mansour et al. The other 5 patients who had temporalis fascia grafting, though there was no graft failure, however retraction (Grade I and II) was seen in 2 cases.

Poe et al reported success of 100 % in 39 patients with TCPC graft in excision of postero-superior retraction pocket. A Tragal cartilage-perichondrium graft, used to reduce recurrent retraction pockets, was placed in posterior-superior quadrant of the tympanic membrane. Temporalis fascia graft was associated with recurrence of retraction at levels varying from 5 to 25 %. This led to failure and subsequent perforations [7]. Similarly Xiao-wei [9] et al conducted a study in 102 patients who had undergone tympanoplasty or tympanomastoidectomy . Seventy-four patients (72.16 %) had tragal cartilage-perichondrium composite graft and 27 patients (27.39 %) had conchal cartilage-perichondrium composite graft. Graft uptake was seen in all patients. Kazikdas et al also achieved 95.7 % graft uptake rate in 22 patients using TCPC graft and 75 % graft uptake in 21 patients with temporalis fascia (*p* = 0.059) [10]. Dornhoffer et al found 95 % success rate with TCPC graft and 90 % with temporalis fascia [11].The TCPC grafting technique was found to have higher success rate ( varying from 95 % to 100 %) as compared to temporalis fascia grafts in various tympanoplasty techniques. [12, 13] However Vaidya et al [14] compared 50 patients each who underwent various types of tympanoplasty for eardrum perforation with chronic otitis media using temporalis and cartilage grafts and found TM closure in 100 % of the patients in conchal cartilage group but only in 84 % in the temporalis fascia graft group. They had included twenty-five patients with atticoantral disease, 11 of whom had temporalis fascia grafts and 14 patients had conchal cartilage grafts but however they have not separately mentioned whether the graft failure in the fascia group included those with chronic otitis media - squamous type or not making comparison with ours difficult.

Though our study showed better graft uptake using temporalis fascia graft, there was no stastistical difference in graft up take in these two groups (*p* = 0.433). Therefore, our results are comparable with that of Mansour et al, Kazikdas et al and Dornhoffer et al. The limitation of our study is that number of patients in our study was very small, multiple surgeons with varying degree of experience in middle ear surgery were involved and in addition the follow up period was only 12 weeks.

## Conclusion

Although it appears that graft uptake rate was better when using temporalis fascia as compared to TCPC graft but there was no statistical difference between use of temporalis fascia or tragal cartilage perichondrium in patients undergoing surgery for chronic otitis media - squamous type. A study with a larger study population and a longer follow up is necessary to determine whether cartilage grafting is truly superior to classical temporalis fascia grafting.

## Abbreviations

COM, Chronic otitis media; CSOM, Chronic suppurative otitis media; CWD, canal wall down; CWU, canal wall up; MRM, modified radical mastoidectomy; PSRP, Postero-superior retraction pocket; TCPC, Tragal Cartilage-Perichondrium Composite graft; TF, Temporalis Fascia graft; TM, Tympanic membrane
